# Participatory learning and action to address type 2 diabetes in rural Bangladesh: a qualitative process evaluation

**DOI:** 10.1186/s12902-019-0447-3

**Published:** 2019-11-04

**Authors:** Joanna Morrison, Kohenour Akter, Hannah Maria Jennings, Tasmin Nahar, Abdul Kuddus, Sanjit Kumer Shaha, Naveed Ahmed, Carina King, Hassan Haghparast-Bidgoli, Anthony Costello, A. K. Azad Khan, Kishwar Azad, Edward Fottrell

**Affiliations:** 1 University College London Institute for Global Health, London, UK; 2 Diabetic Association of Bangladesh, Dhaka, Bangladesh

**Keywords:** Behaviour change, Qualitative, Non-communicable disease, Diabetes, Process evaluation, Randomised controlled trial, Theory of change

## Abstract

**Background:**

Diabetes is 7th largest cause of death worldwide, and prevalence is increasing rapidly in low-and middle-income countries. There is an urgent need to develop and test interventions to prevent and control diabetes and develop the theory about how such interventions can be effective. We conducted a participatory learning and action (PLA) intervention with community groups in rural Bangladesh which was evaluated through a cluster randomised controlled trial. There was a large reduction in the combined prevalence of type 2 diabetes and intermediate hyperglycaemia in the PLA group compared with the control group. We present findings from qualitative process evaluation research to explore how this intervention was effective.

**Methods:**

We conducted group interviews and focus group discussions using photovoice with purposively sampled group attenders and non-attenders, and intervention implementers. Data were collected before the trial analysis. We used inductive content analysis to generate theory from the data.

**Results:**

The intervention increased the health literacy of individuals and communities - developing their knowledge, capacity and self-confidence to enact healthy behaviours. Community, household and individual capacity increased through social support and social networks, which then created an enabling community context, further strengthening agency and enabling community action. This increased opportunities for healthy behaviour. Community actions addressed lack of awareness about diabetes, gendered barriers to physical activity and lack of access to blood glucose testing. The interaction between the individual, household, and community contexts amplified change, and yet there was limited engagement with macro level, or ‘state’, barriers to healthy behaviour.

**Conclusions:**

The participatory approach enabled groups to analyse how context affected their ability to have healthy behaviours and participants engaged with issues as a community in the ways that they felt comfortable. We suggest measuring health literacy and social networks in future interventions and recommend specific capacity strengthening to develop public accountability mechanisms and health systems strengthening to complement community-based interventions.

**Trial registration:**

Registered at ISRCTN on 30th March 2016 (Retrospectively Registered) Registration number: ISRCTN41083256.

## Introduction

Globally, an estimated 422 million people have diabetes and 75% of those are in low- and middle-income countries [1]. In 2016, diabetes was the 7th largest cause of death, with 1.6 million deaths attributed directly to diabetes [2]. The United Nations Sustainable Development Goals acknowledge that many of these deaths were preventable and premature, and targets have been set to reduce premature mortality from non-communicable diseases (NCDs) by one third by 2030. The WHO action plan for NCDs recognises the need for a multi-sector approach which includes empowering communities and meaningfully engaging civil society to raise awareness, educate, advocate and monitor progress [3].

A collaboration between the Diabetic Association of Bangladesh and the University College London Institute for Global Health conducted a qualitative process evaluation (PE) [4] of a community mobilisation intervention of groups practicing participatory learning and action (PLA) [5–7]. The PLA intervention was inspired by the philosophy of Paulo Freire. He believed that development of a critical consciousness about a shared context among marginalised groups was a precondition for positive behaviour change [5, 6]. When groups of people develop critical consciousness, they apply critical thinking skills to discussions about their communities, how community conditions affect them, and how they can take action to improve their lives and their communities [8]. Freire’s philosophy has been systematized in a cycle of problem identification, planning together, implementation and participatory evaluation [6].

The PLA intervention was part of a three-arm cluster randomised controlled trial to test the effectiveness of mobile phone messaging (mHealth), and PLA groups compared with control areas on the prevalence of intermediate hyperglycaemia and type 2 diabetes and two-year cumulative incidence of diabetes among an intermediate hyperglycaemia cohort [9]. There were 122 PLA groups with an average of 27 group members. There was a 20% absolute reduction in diabetes and intermediate hyperglycaemia prevalence and 10% reduction in the two-year cumulative incidence of diabetes among the group with intermediate hyperglycaemia in the PLA versus control arm, and the intervention was highly cost-effective. There was no effect of PLA on blood pressure, overweight and obesity, or self-reported physical activity or fruit and vegetable consumption, and further analysis is ongoing [10].. We conducted a process evaluation to 1) describe how the intervention was implemented 2) explore fidelity to the participatory approach [11] and 3) to develop the theory about how PLA interventions can prevent and control diabetes. PLA interventions are complex, having dynamic components which interact with individual, household and socioenvironmental risk factors to affect behaviour [12]. Therefore, we have used an ecological approach [13–15] to develop the theory about the mechanisms of how PLA groups were effective in addressing type 2 diabetes.

The ecological approach is a broad, overarching paradigm which refers to the study of individual and environmental determinants of behaviour, and their interactions. It is often represented diagrammatically by a series of nested circles representing different spheres of influence [13, 14]. It rejects an oversimplification of person-focused approaches to public health problems, and instead acknowledges their complexity [16]. The ecological approach emphasises the role of social context in modifying public health conditions and mediating mechanisms of change [15, 17], and recognises that the relationship between the individual and context is dynamic, multidirectional and multilevel [18].

## Materials and methods

### Setting

The intervention was implemented in four upazillas in Faridpur in central Bangladesh from July 2016 to December 2017. An upazilla is a geo-political sub-unit of a district. We collected process evaluation data in all intervention upazillas in January 2018, before the trial analysis. Faridpur is around 2000 km^2^ and has a population of over 1.7 million, with a predominantly agricultural economy of jute and rice farming. The population is mainly Bengali, as is the case in most of Bangladesh. Almost 90% of the population in Faridpur are Muslim, with Hinduism being the second largest religion [19]. Bangladesh is a patriarchal society, with patrilocal marital practices where women come under the guardianship of their husband’s family after marriage [20]. Moreover, in Bangladesh, *purdah* (female seclusion), restricts women’s freedom of movement and their access to public spaces. Women are expected to behave demurely, avoid attracting attention from men, and uphold family honour [21]. Norms about the division of labour within and outside the household, as well as norms about movement outside the household are highly gendered [22].

A baseline survey found that over 70% of men and women reported at least three risk factors for type 2 diabetes. Women were more likely to be overweight or obese and spend less time doing physical activity at a younger age than men. Less than 30% of women aged 70 years or more had adequate levels of physical activity. Men in all wealth groups had higher levels of tobacco use than women, with 63% of men currently using tobacco compared to 41% of women. Women were more likely to chew tobacco or use snuff than men. Consumption of white rice, fried food and sweets was common among both men and women. Only one in three respondents could report any causes of type 2 diabetes, and only 14% of respondents had had a blood glucose test, despite high prevalence of intermediate hyperglycaemia and type 2 diabetes (men: 17.2 and 8.9%; women: 23.4 and 11.5%) [23, 24].

In the study area, diagnosis and management of diabetes was only possible at two diabetic hospitals run by the Diabetic Association of Bangladesh in urban areas of Madhukahli and Faridpur, and three upazilla health complexes (government hospitals). Community Clinics and Family Welfare Centres at village level should be able to screen for diabetes and refer but, in practice, blood glucose testing facilities were not routinely available. Informal health workers and drug vendors were often used to monitor diabetes.

### Sampling

We conducted process evaluation (PE) research in 15 intervention villages. We requested supervisors of PLA groups to identify groups which had been most active in implementing communication action to prevent and control diabetes. We sampled in this way because we were limited in time and resources and sought to explore how the intervention could be effective in an optimal context. We asked PLA group facilitators to locate four different types of participant across the 15 villages who were then invited to discussions in these villages. We purposively sampled 1) men and women who were over 30 years old, had regularly attended PLA group meetings, and were interested to participate, 2) men and women with diabetes who were over 30 years old, regularly attended PLA group meetings, and were interested to participate and 3) PLA group facilitators. We conveniently sampled 4) men and women who did not attend PLA groups. The PE manager (KAk) facilitated discussions to explore how participants and communities had experienced the intervention.

### Data collection

KAk conducted four group interviews (GI) with male attenders, four GIs with female attenders, one GI with male diabetic attenders, and one GI with female diabetic attenders. She conducted one GI with a male non-attender, and one GI with a female non-attender (Table 1). GIs had a similar format to a semi-structured interview, and conversation occurred primarily between the researcher and the participant, as opposed to between participants [25, 26]. GIs had 3–5 participants and we used participatory photography, or ‘photovoice’ [27], and discussion to explore personal and group experience of the intervention. Photovoice typically entails giving participants cameras and inviting them to take photos which represent issues of importance to them. The photographs and the narrative about the issues they identify can facilitate communication about an issue within and between groups of people. Typically, photovoice has been used in participatory action research with marginalised groups to identify and communicate about their experience of social problems in order to stimulate action to address problems [28, 29]. To a lesser extent, it has also been used in evaluation research [30–32]. Photovoice enables greater participant control over the research process [28, 29, 33], and enables critical reflection prior to discussion with researchers [34]. This can increase participant confidence and facilitate better communication between researchers and participants [35]. KAk took four mobile phones with cameras to each group and asked them to take photos that illustrated how the intervention had affected them, their family and community. If they could not take photos of the subject they wanted to, they should take photos of a representation of that subject. Participants discussed if they wanted to take photos individually, or together, and how to share cameras. After they had taken photos, they informed KAk who arranged a convenient time and place to conduct the GIs. Photos were printed and given to participants at discussions.

**Table 1:** Data Collection

Respondent type & method	Upazilla & participants			
	Boalmari (n)	Nagarkhanda (n)	Saltha (n)	Madhukhali (n)
Male attender group discussion (GI)	1 (5)	2 (8)	2 (9)	
Male attender focus group discussion (FGD)			2 (15)	
Male non-attender		1 (4)		
Female attender GI	1 (4)	2 (9)	1 (5)	1 (3)
Female attender FGD	1 (9)		2 (20)	1 (7)
Female non-attender				1 (5)
Total	**3 (18)**	**5 (21)**	**7 (49)**	**3 (15)**

KAk also conducted two focus group discussions (FGDs) with 6–8 male attenders, and four FGDs with 6–8 female attenders to explore how the intervention had affected communities and explore triangulation with the data collected through other methods. KAk took observation notes during and after field visits. She used topic guides in GIs and FGDs to explore the barriers and enablers of behaviour change and how the intervention was able or unable to address these. The topic guide and photovoice process were piloted in one group discussion with non-diabetic men and, after minor adjustments, we felt that further piloting was not necessary. Discussions ranged from 35 min to 2 h and 5 min. KAk took informed consent from all participants and informed consent to show the photos in public forums. Participants also took informed consent from those who were the subject of the photograph.

### Data management and analysis

FGD data were digitally recorded and KAk made notes about the findings in English. She translated field observation notes to English. Other data were digitally recorded and directly transcribed and translated into English. A sample of translated transcripts were checked against the recordings by KAk. Photos were inserted in transcripts and analysed with verbatim data. Transcripts were imported into Nvivo for analysis. We used inductive content analysis to generate theory from the data [36]. Data were analysed by KAk who is Bangladeshi and bilingual, HMJ who is fluent in Bangla and has lived in Bangladesh for several years, and JM who has lived in and conducted research in South Asia for over 10 years. JM and HMJ familiarised themselves with the data and listed the key themes independently before discussing these together with KAk and conceptualising a draft analytical framework. This enabled us to be reflexive and check our possible bias. This framework was applied to four transcripts by JM and adapted before all data were coded in Nvivo. JM compared data from the different types of respondents, looking for patterns, and developed a description of the main findings which she discussed with KAk, HMJ and other members of the trial team. Finally, we compared our findings with mixed methods longitudinal PE data (reported elsewhere) to explore triangulation and enhance the validity of our study [11].

## Results

### Individual and group level mechanisms

#### Health literacy and self-confidence

Diabetic and non-diabetic attenders felt that group discussions helped develop their individual knowledge about the causes, symptoms and complications of diabetes, and how to prevent, test for and control diabetes: “I ate fried snacks a lot before and enjoyed eating them, but after I found out that they are unhealthy I do not buy them.” (Attender women 020FGD). Individual knowledge about diabetes risk factors was developed among group members: “Before we didn’t have any idea about exercising. We didn’t know that diabetes could be controlled through exercise. We learnt that from the meeting. We also didn’t know that we should eat more vegetables. We used to eat more rice. I was also like that.” (Attender men 007GI).

Men and women attenders reported that the group discussion, reiteration, and participatory methods made it easier for them to understand information. Individual attenders spoke to each other about topics raised in the group which re-confirmed what they had learned and helped them feel confident in this knowledge: “We get together here, gossip and share with each other, which we enjoy. We discuss the meeting topics when we have free time and go for a walk. We are illiterate. It is not possible for me to read so when someone tells me something then it is easier for me to remember.” (Attender women 003GI). This confidence enabled attenders to tell others about what they had learned, explain changes in their behaviour, and persuade more individuals, families and community members to behave differently: “Many people bathe in our pond. I went there and said: ‘If we swim, we can prevent diabetes’. Many people became aware and now they will also spread this knowledge.” (Attender men 007 GI). Having attended a group, attenders felt that the credibility of their advice increased: “As a group, people listen to us if we tell them something. But if we were not in a group and we told them something, people would consider us crazy and they wouldn’t listen to us. When a group of people do something, other people take it differently and try to follow the advice.” (Attender men 001GI). Non-attenders reported learning about how to prevent and control diabetes through talking with attenders, PLA group facilitators, and from attending the community meeting – a one-off large meeting to disseminate knowledge about group activities and plan community-wide actions: “We went there and learnt some issues from that (community) meeting … they said to take less rice and more vegetables, lemon, and green pepper.” (Non-attender women 009 GI).

#### Social support and positive reinforcement

Men and women discussed their success in changing their behaviour - exercising more, reducing salt, oil, and sugar consumption, eating less rice and sweets, eating more vegetables and reducing tobacco intake. These changes were enabled by social motivation from facilitators, group attenders, family members, informal health workers, health workers and local leaders. Positive reinforcement, and an enabling family and community environment also supported change.

Men and women attenders and non-attenders were motivated by each other. Group physical activity and introducing a competitive element encouraged behaviour change: “This is the pond where we swim (shows photo). We had a swimming competition here. Swimming is an alternative to walking and it is good exercise. Sometimes we compete while swimming. We all swim together. My weight has decreased now because since attending the meeting I have started walking and swimming.” (Attender women 006GI).

Men and women felt positive and optimistic about these changes and reported multiple benefits. Many felt that they had taken control of their health: “We found that if we maintain the rules that we learned at the meeting we feel good. So, we don’t need to go to the doctor, which saves us money. I like this the most. I reduced my consumption of oil and rice, and I’m growing vegetables and eating more of them. All of this saves us money and keeps us physically well. If the group didn’t exist, we would not be able to learn about these issues which we didn’t know about before, and so we might be affected by diabetes one day.” (Attender men 002GI).

### Household and social contextual mechanisms

#### Agency, resilience & changed social norms

##### Physical activity

Our formative research showed that before the intervention, women often felt unable to walk for exercise because they were not allowed by their husbands, they were too embarrassed, they felt unsafe, or they were afraid of social criticism [21]. Men and women were also afraid that people would think they had diabetes if they walked. Men’s and women’s groups used dramatic representation of these stigmas at community meetings which enabled discussion and initiated action for social change. Many men encouraged and gave explicit permission for their wives to walk, and many agreed not to criticise women: “The main problem of walking was embarrassment. Women did not feel comfortable to walk outside. They were also concerned about men and older people disliking this, and men would criticise and make fun of them. When we discussed this issue in the community meeting men said that they would not criticise anymore.” (Attender women 019FGD). Not all men were supportive, but women reported a change in men, and in the social environment. Women who walked to a neighbouring village noted that they were criticised by men who were unaware of group activities: “We need to go far, to the next village, and men make comments as they do not have knowledge like in our (intervention) village.” (Attender women 019FGD). While some participants were still concerned about criticism and stigma, group activity helped them feel more resilient and it was now more socially acceptable to walk. Men said: “It was very common before that if anyone walked people asked them why they were walking … .no one asks anything now because they know the reason for walking.” (Attender men 018FGD).

##### Diet

Group activities and social change made it easier to eat healthily while socializing in intervention areas: “People used to pressure a guest to eat more but now they don’t do this. Now, the rule is that you eat by your own choice.” (Attender men 021 FGD). More men than women reported this change. Both male and female attenders stated that they were less afraid of being called ‘miserly’ or ‘poor’ because of their healthier eating habits.

#### Increasing opportunities for healthy behaviours

##### Household

Vegetables were perceived to be expensive, polluted with chemical fertilisers and pesticides and inconvenient to buy. Vegetables were not always available locally and had to be bought frequently to increase the intake suggested at meetings. Men usually shopped for food and women reported that they were “irritated with them asking to bring spinach every day.” (Attender women 020FGD). Therefore, households focused on growing their own vegetables. Attenders and non-attenders took photos of their kitchen gardens illustrating that they had increased their access to vegetables through growing more at home. Although some had grown vegetables previously, they reported realising the importance of home cultivation: “This is the picture of gourd plant. I planted this before. But after I went to the meeting, I thought ‘this is important’, as I heard that we should eat more vegetables and less rice. Planting gourd helps my family eat more vegetables.” (Attender men 002GI). Many participants took photos of their home farming and said that milk, eggs and meat sales enabled them to buy fruit, vegetables, and fish, and pay for blood glucose testing and medicine.

Household interaction also enabled healthy eating behaviours. Many women described getting the whole family used to the taste of food with less oil. They also withdrew opportunities for unhealthy behaviour and discussed the reasons for the change in taste: “Firstly, we faced some problems regarding cooking. It was tasteless. For example, un-ripened banana or potato curry – these were tasteless … if we cooked with less oil, our husbands found that food tasteless. Initially it was difficult to eat, but after a while we got used to it … now, our husbands do not accuse us of anything as they have also learned … we realized that we need to follow the rules and use less oil to stay healthy and they now agreed with us and listen to us … if we use less oil then it will also save money (laughing).” (Attender women 004GI).

##### Community

Men’s groups increased opportunities for healthy behaviour in the community. For example, some local shops began to sell healthier snack foods, such as eggs, puffed rice and bananas: “I used to eat fried snacks in the afternoon. Now I avoid that. The shop now sells boiled eggs. We suggested them to do that because I don’t eat fried snacks.” (Attender men 007GI). Another group addressed smoking in public places: “We suggested to people not to smoke as it’s bad for their health. We prohibit people from smoking in the tea stall.” (Attender men 007GI).

#### Increasing opportunities for care-seeking

##### Increasing access to resources

Many group attenders wanted to save money through the group. Women were particularly keen to do this, as they had few opportunities to save. Forty-three groups initiated a fund which was often used for medical treatment. Funds were broadly perceived to be a form of social support: “In my group one person has taken money for treatment of a kidney problem ... In my group some have borrowed money for a check-up and they have repaid it … A few women have taken money to buy vegetables in my group.” (Facilitator FGD). Some groups had also used the fund for blood glucose testing, which was an action agreed upon at the community meeting.

##### Increasing demand and supply of health services

The opportunity costs to reach facilities and the actual cost of care were prohibitive to care-seeking. Social norms dictated that women should be accompanied while travelling which made their access to care particularly challenging. The group and community meetings increased demand for blood glucose testing. Increased knowledge about prevention and control of diabetes also reduced the fear of a diagnosis and fear of resultant stigma, costs and lifestyle changes, which had prevented some people from being tested. Groups interacted with local politicians and service providers to improve access to services but were unsuccessful. Instead groups in 32 villages arranged for an informal health worker to visit the village to conduct blood glucose testing. 74 groups organized local blood glucose testing twice during the intervention period: “I never got tested before because I was scared that I would be tense … I have now checked here in the group meeting once and twice in the market. I also told my wife to take care and check for diabetes. Me and my wife have both been checked.” (Attender men 012 GI). The awareness created by the group meant that more female participants felt comfortable in asking for support to seek care. Increased awareness among male family members, and an expanded social support network enabled the creation of a supportive environment for care-seeking. Women said: “Now, we do not have a problem to seek care. We get help from our family members if we feel ill. Family members also insist that we check for diabetes or go to the doctor or health facility.” (Attender women 020 FGD).

## Discussion

There is a need to move away from individualistic, linear approaches to behaviour change and acknowledge the role of structural and systemic factors, social habits, and socio-cultural influences on behaviour [37, 38]. Increasing knowledge and risk understanding alone is unlikely to result in health-related behavioural changes if individuals continue to experience structural constraints. In taking a PLA approach informed by Paulo Freire [5, 8], we sought to catalyse the development of a critical consciousness through group dialogue, active education, and participatory action to develop community driven responses to diabetes.

**Fig. 1:**
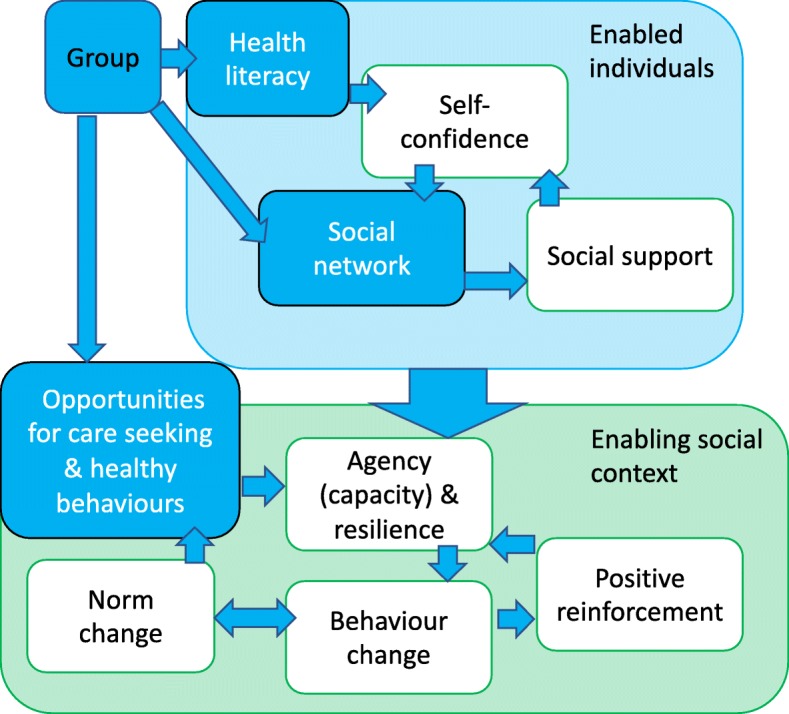
PLA Behaviour Change Mechanisms

Participants reported that the group and group activities stimulated change at individual, household and community levels. The intervention was successful at increasing the health literacy of individuals and communities - developing their knowledge, capacity and self-confidence to enact healthy behaviours. Community, household and individual agency (ability to enact behaviours) increased through social support and social networks. This then created an enabling community context which further strengthened agency and enabled community action to be taken, which increased opportunities for healthy behaviour. The interaction between the individual, household, and community contexts enabled, reinforced and amplified change (Fig. 1). We discuss the foundational mechanisms of the intervention: health literacy, social support and increased opportunities for healthy behaviour.

### Health literacy

The ‘active education’ component of PLA was important in increasing health knowledge and confidence, which stimulated other change mechanisms. It is important to differentiate health literacy from health knowledge, as this inadequately conveys the cognitive skills and embedded understanding which develops in becoming health literate [39]. Health literacy is defined as the personal, cognitive and social skills which determine a person’s ability to gain access to, understand, and use information to promote and maintain good health [40]. Low levels of health literacy are associated with worse health outcomes for persons with diabetes [41–43], and therefore it is an important intervention target area. Group members felt enabled to change their behaviour because they had learned and become confident. Some attenders had low levels of functional literacy, and yet our data show that they demonstrated communicative, interactive and critical literacy in their ability to extract information and use it to exert control over life events and situations [40]. Participants attributed this change to the intervention and to the participatory methods which engaged group members in critical thinking through problem-posing and dialogue. Our intervention was also in congruence with published strategies to improve health literacy, such as limiting the amount of information to key messages, avoiding jargon, and using pictures to explain concepts [44]. Future research could determine the suitability of health literacy measurement tools (such as [45]), before adapting and applying them to evaluate how health literacy mediates changes in health outcomes.

### Social networks and social support

Social relationships impact on physical and mental health [46]. Research on family and social support and network interventions has shown positive effects on self-management of diabetes in high-income countries [47–49] and risk factors [50]. Our study differs from these interventions, as it engaged the general population and sought to address several diabetes risk factors. The social networks which were established or activated through the PLA intervention were organic and enabled the provision of emotional support (warmth and commitment), appraisal support (helping a person understand an event and the resources that could help deal with the situation) informational support (advice and information), and tangible support (material or practical help) [51]. Participation in a community-based organisation has been associated with better health in persons with diabetes [52]. Our study shows that group participation and group action can increase social support networks beyond the group into the family and social context. It will be important to collect and analyse data on social networks and social support in future PLA interventions, particularly because they may play an important role in the sustainability of the intervention and its effects [53]. Future research could explore in more depth how social support interacts with other pathways of change [54, 55], and how the type of network and the way that it works affects pathways of behaviour change [51, 56].

### Action to increase opportunities for healthy behaviour

We found that there was some engagement with structural constraints to healthy behaviour. For example, groups pressured men and communities to support women who walked to prevent or control diabetes and pledged not to tease or criticise physical activity. Other actions acknowledged structural constraints and took a pragmatic approach to addressing them. For example, gender norms limited movement of women preventing their access to vegetables, and groups addressed this by having renewed energy and commitment for kitchen gardening. After a few failed attempts to address the lack of blood glucose testing facilities in government health facilities, groups organised and paid for community level private testing. Our intervention was implemented over a relatively short period of time, and group members may have sought to exhaust local solutions before engaging with state systems. Alternatively, they might have felt unwilling or unable to demand systems change, perhaps lacking in confidence that they could achieve change [57]. Community participatory approaches have been criticised for devolving responsibility to address health problems from the state to local communities [58]. While we do not fully agree with this criticism, our findings suggest the need for public health policy responses to non-communicable diseases to complement community based efforts [59, 60]. It will be important to evaluate the effectiveness of group-initiated strategies to maintain behaviour change over time, and the possible amplification effect of complementary systems interventions.

### Limitations

We used group facilitators to identify participants for the study, because the group intervention had ended, which may have biased sampling towards recruiting active attenders. Attenders may have been motivated to respond positively because they sought to secure continued support for the intervention, but we also found that data from non-attenders was overwhelmingly positive. The PE manager (KAk) spent substantial time in Faridpur observing the intervention during the trial and collecting longitudinal PE data. We compared our findings with longitudinal data and found there was a high degree of triangulation, indicating the validity of our findings. Data collection and analysis were undertaken before the trial analysis, to generate hypotheses [61]. While writing this article, the trial results were made public, which may have biased our interpretation of the data.

## Conclusion

The PLA intervention improved health literacy, promoting change at individual, household and community levels. Engagement with health systems or the state was limited, perhaps because of the relatively short intervention timeframe. The participatory approach allowed groups and community members to analyse how their context affected their behaviour, and enabled community engagement and actions which were feasible and acceptable. We found that the interaction between the individual, household, and community contexts enabled, reinforced and amplified change. Complex mechanisms of effect shown here do not lend themselves easily to quantitative measurement, however further study of social networks and health literacy may be possible in future research. Given the increasing global burden of non-communicable diseases it is important to develop and test interventions that are contextually relevant and explore their mechanisms of effect. We have shown that community-based PLA interventions are an effective and cost-effective approach to addressing diabetes in rural Bangladesh and would be complemented by systems strengthening.

## Data Availability

Data is available from the corresponding author subject to the approval of the UCL/BADAS data sharing committee, which will assess requests on a case by case basis.

## Abbreviations

BADAS: Diabetic Association of Bangladesh
FGD: Focus Group discussion
GI: Group Interview
PE: Process evaluation
PLA: Participatory Learning and Action
